# Treatment of snoring using a non-invasive Er:YAG laser with SMOOTH mode (NightLase): a randomized controlled trial

**DOI:** 10.1007/s00405-022-07539-9

**Published:** 2022-07-22

**Authors:** Valerie A. Picavet, Marc Dellian, Eckard Gehrking, Alexander Sauter, Katrin Hasselbacher

**Affiliations:** 1 ENT, Praxis für Ästhetik/HNO, Ludwigstrasse 7, Augsburg, Bavaria Germany; 2 ENT, MVZ Moser Gehrking Sauter und Partner, Augsburg, Bavaria Germany; 3 ENT Praxis Hasselbacher Picavet & Partner, Donauwörth, Bavaria Germany

**Keywords:** Snoring, UARS, OSA, NightLase

## Abstract

**Objectives:**

The aim of this study was to assess safety and efficacy of a non-invasive 2940 nm Er:YAG treatment with SMOOTH mode in reducing snoring in adult patients and to compare its efficacy and safety to sham treatment in a randomized controlled trial setting.

**Methods:**

40 primary snoring patients (≥ 18 year, AHI < 15e/h, BMI ≤ 30) were randomized to receive either 3 sessions NightLase or sham laser treatment. The main outcome measures were Snore Outcomes Survey (SOS), the Spouse/Bed Partner Survey (SBPS), a visual analogue snoring scale (bed partner) and a visual analogue pain scale.

**Results:**

NightLase was well tolerated, no local anaesthesia was required (mean VAS pain score in NightLase group = 3.0 ± 1.7). No complications occurred. SOS, SBPS and VAS snoring scores improved in the NightLase group (33.7 ± 14.1 to 56.2 ± 16.1) (35.0 ± 17.1 to 61.5 ± 16.4) and (7.9 ± 2.0 to 4.7 ± 2.8) while no changing in the sham group (32.2 ± 14.5 vs 32.1 ± 13.0) (36.7 ± 12.1 vs 34.7 ± 12.7) (8.1 ± 1.7 vs 8.0 ± 1.6), respectively.

**Conclusions:**

NightLase is a safe, minimal invasive treatment that significantly reduced snoring compared to sham treatment.

## Introduction

Snoring occurs as a result of soft tissue vibration caused by a partial upper airway collapse during sleep [1]. For the treatment of snoring, lasers are traditionally used in an ablative way to reduce soft tissue hypertrophy. Recently, a non-ablative laser modality using an Er:YAG laser with non-contact SMOOTH mode (NightLase by Fotona) has been shown promising in treatment of snoring and apnea [2–7]. It involves an easy to perform, patient friendly non-ablative heating of the oropharyngeal tissue that requires no special preparation, anaesthesia, or post-treatment therapy.

The patented Smooth mode consists out of a series of sub-ablative micro pulses. These very short temperature pulses, as generated at the epithelial surface, are then transformed via heat diffusion into a long lasting thermal pulse within the deeper lying connective tissue. As a result, two complementary regenerative processes are initiated: (1) an indirect triggering effect by short duration heat shocking of the epithelium and (2) a direct slow thermal injury of the connective tissues. Both result in collagen remodelling and neocollagenesis. Consequently, the oropharyngeal mucosa is strengthened and its vibration capacity and collapsibility is reduced. By that, an expansion of the pharyngeal airway is achieved. The histological effects of NightLase were shown by Unver et al. in the soft palate of rats [8]. They found a shrinkage of the palatal mucosa with no evidence of bleeding, severe inflammation, carbonisation or necrosis. In addition, a pilot study by Lee et al., showed, that the photothermic effects of NightLase significantly increased the airway volume and the minimal cross-sectional area at 12 weeks post-laser treatment, measured with three-dimensional imaging of the upper airway using Cone Beam Computed Tomography (CBCT) [3].

Taking into account the minimal invasivity of NightLase, it can represent a good alternative to more aggressive treatment options for snoring. The aim of this study was to evaluate the efficacy of a non-invasive 2940 nm Er:YAG in reducing snoring and to compare its efficacy and safety to sham treatment in a randomised controlled trial setting.

## Materials and methods

The study protocol was approved by the ethics board of the Faculty of Clinical Medicine of the Ludwig-Maximilians-University Munich, Germany.

### Patient selection

Adult patients (≥ 18 years) with primary snoring/mild OSA (AHI < 15e/h as measured with home respiratory polygraphy and/or polysomnography), a maximum BMI of 30 and without daytime sleepiness (ESS ≤ 9) were included if they had complained about socially disturbing snoring and asked for treatment. Both non- and invasive treatment alternatives including weight reduction, positiontherapie, intraoral devices, laser-assisted uvulopalatoplasty and UPPP were offered to all our patients. Nasal obstruction and tonsil hypertrophy as a cause for snoring were excluded.

Patients that fulfilled the inclusion criteria and signed the informed consent form were randomised using the online randomisation software Sealed Envelope (www.sealedenvelope.com) to either laser or sham treatment.

### Laser procedure

The oropharynx (soft palate, anterior and posterior tonsillar pillar, tonsils, uvula and base of the tongue) and the posterior part of the hard palate were treated with 2940 nm Er:YAG laser wavelength in a non-ablative, thermal SMOOTH™ mode (implemented in SP Dynamic laser system, Fotona, Slovenia) using a non-contact PSO3 hand-piece with 7 mm spot size and collimated laser beam. The latter permits the practitioner to move the hand-piece over a range of positions without significantly defocusing or altering the spot size of the beam. All procedures were performed by the same MD. The laser parameters were set to the following: SMOOTH Mode with a fluence of 8.5–9 J/cm^2^ with 1.6–2.2 Hz performing 4–6 smooth pulses per spot and total smooth pulses ranging between 2011 and 2297 per session (Table 1).

**Table 1:** Lasersetting NightLase SP Dynamis, Fotona

	Erb:YAG
Handpiece	PSO3X
Spotsize	7 mm
Fluence	8.5–9 J/cm^2^
Pulse Mode	SMOOTH mode
Frequency	1.6–2.2 Hz
Stacks/Spot	4–6 SMOOTH Puls/Spot
Overlap	No Overlap
Number of Pulses	2000–2300 SMOOTH Pulses

Each patient underwent 3 treatments in a period of 42 days (approximately at days 0, 14–21 and 42). Patients in the sham group were treated using the same laser but with an attached sham hand-piece, which blocks the passage of the laser light onto the tissue. In that way, patients remained blinded, as they see and hear the device in the active mode.

### Study outcomes

*Snore Outcomes Survey (SOS)—*The SOS is a reliable and valid instrument for assessing sleep-related health status for patients with snoring and sleep-disordered breathing and for measuring change in health status following therapy.

It consists of 8 items relating to the intensity, duration, frequency and impact of sleep disordered breathing symptoms—specifically snoring [9]. Because of the impact of Sleep disordered breathing (SDB) on the bedpartner, a separate *Spouse/Bed Partner Survey (SBPS*) containing 3 Likert-type items was also included. Scores on the SOS and SBPS are normalized on a scale ranging from 0 (worst) to 100 (best) [9].

A *visual analogue snoring score* (0 no snoring—10 extreme snoring/sleep separately) to evaluate snoring severity by the bed partner.

Patients and their bed partners were asked to fill out the above mentioned questionnaires before treatment, after each laser session and 3 months after the last procedure.

In addition, immediately after every session, and at days 1 and 3 after treatment, patients were asked to mark the perceived pain on a *visual analogue pain scale* (0 = no pain, 10 = worst pain) and to rate specific side effects at days 1 and 3: sore throat (no-very mild–mild–moderate–severe), disturbed taste (no-very mild–mild–moderate–severe), foreign body sensation (no-very mild–mild–moderate–severe). If moderate to severe symptoms occurred, patients were asked to contact us, to check for mucous damage or aphthous ulcer formation.

### Statistics

The sample size was determined according to the assumption that the NightLase treatment is an effective treatment for snoring and was derived from the effectiveness data from previously published studies [4, 5], taking the average drop in the VAS snoring score, according to bed partners, into account. From the randomized controlled trial of a placebo-controlled trial of radiofrequency surgery for snoring, it was estimated that the placebo effect on the VAS snoring score evaluated by the bed partner was minimal [11]. Based on these values, it was estimated that 16 patients in each group will be sufficient to prove NightLase effectiveness over the sham treatment with 80% statistical power. Accounting drop-out rate of 15–20%, 20 patients were recruited to each arm. Changes in SOS, SBPS and VAS snoring were calculated. Statistical analysis comparing the effectiveness between laser and sham group was performed up to the 3-month follow-up after last treatment (two-sample rank sum, *U* test). *P* values < 0.05 were considered statistically significant. Statistical analysis was performed using SAS 9.4 (SAS Institute).

### Results

The NightLase group consisted out of 20 patients, 7 women and 13 men, with a mean age of 43.3 ± 10.1 years (range 24–67) and mean BMI off 26.6 ± 3.6. All patients in the NightLase group completed the protocol. One patient in the NightLase group was excluded at the 3-month follow-up because of a change in weight ≥ 5 kg. The Sham group consisted out of 20 patients, 5 women and 15 men, with a mean age of 44.5 ± 8.3 years (range 33–62 years) and mean BMI off 26.9 ± 3.9. In the sham group, 5 patients dropped out after the second treatment (Table 2).

**Table 2:**
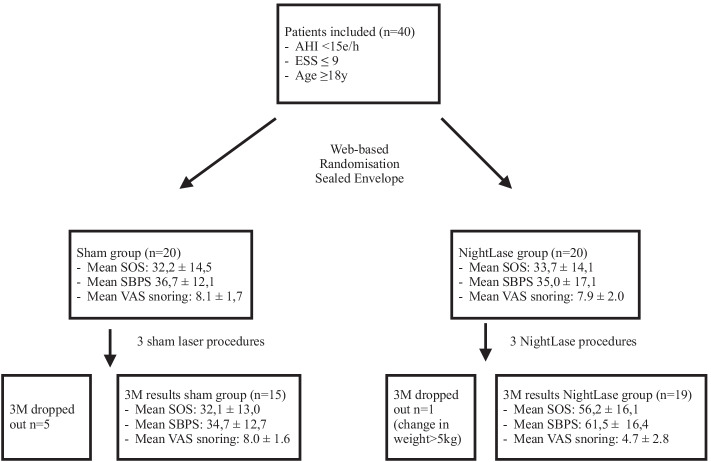
Study flowchart—results

In none of the patients local anaesthesia was needed. Mean VAS pain score in the NightLase group was 3.3 ± 1.9 at time of treatment, 0.3 ± 0.5 at day 1 and 0 at day 3. All the observed side effects were mild and transient. At day 1, 4 patients reported a very mild sore throat (20%), one patient a mild sore throat (5%), and 3 patients a very mild sensation of dry throat (15%) and/or a very mild foreign body sensation (15%). None of the patients reported a disturbed taste. All symptoms had diminished at day 3. In none of the patients ulceration or scarring occurred.

**Fig. 1:**
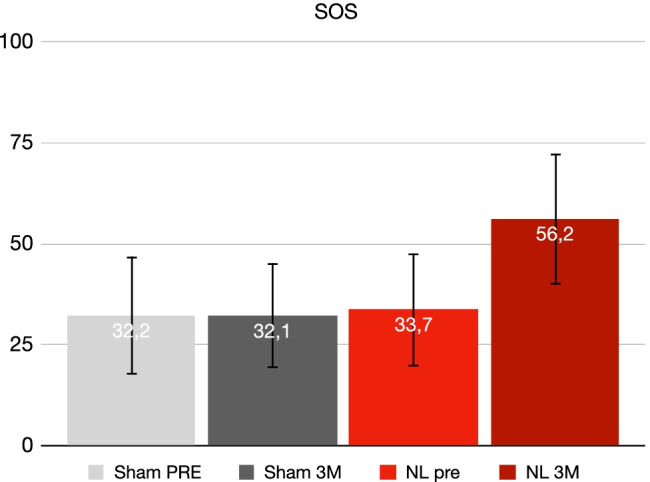
Mean SOS scores remained unchanged in the sham group (32.2 ± 14.5 vs 32.1 ± 13.0), while improved in the NightLase group from 33.7 ± 14.1 to 56.2 ± 6.1 at 3 M follow up

**Fig. 2:**
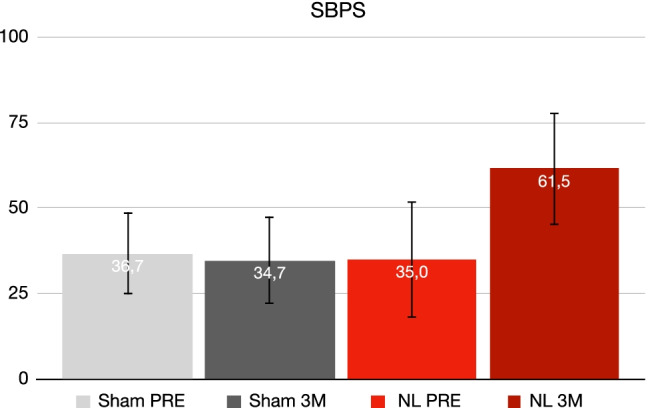
Mean SBPS scores remained unchanged in the sham group 36.7 ± 12.1 vs 34.7 ± 12.7, while improved in the NightLase group from 35.0 ± 17.1 to 61.5 ± 16.4 respectively at 3 month (M) follow up

**Fig. 3:**
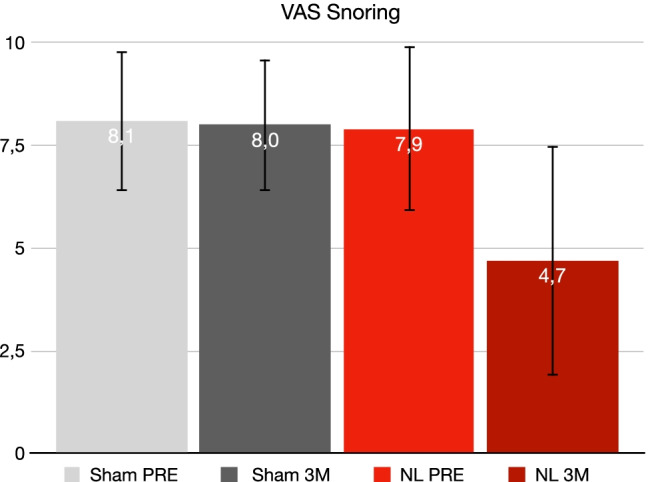
Mean visual analogue snoring scores as assessed by the bed partner remained unchanged in the sham group 8.1 ± 1.7 vs 8.0 ± 1.6, while it improved in the NightLase group from 7.9 ± 2.0 preoperative to 4.7 ± 2.8 at 3 month (M) postoperative

Mean SOS and SBPS scores remained unchanged in the sham group (32.2 ± 14.5 vs 32.1 ± 13.0 and 36.7 ± 12.1 vs 34.7 ± 12.7, respectively), while improved in the NightLase group from 33.7 ± 14.1 to 56.2 ± 16.1 and from 35.0 ± 17.1 to 61.5 ± 16.4, respectively, at 3 M follow-up (Figs. 1, 2). Similarly, mean visual analogue snoring scores as assessed by the bed partner remained unchanged in the sham group (mean VAS snoring score before surgery: 8.1 ± 1.7 vs 8.0 ± 1.6 after surgery), while it improved in the NightLase group from 7.9 ± 2.0 preoperative to 4.7 ± 2.8 postoperative (Fig.  3). There was a statistically significant difference between the two groups regarding the changes in SOS scores, SBPS and VAS snoring (*p* < 0.001).

## Discussion

For the first time, the effectiveness of NightLase was investigated in a prospective, placebo controlled study. We found that NightLase treatment was significantly more effective than sham laser treatment in reducing socially disturbing snoring. In addition, sleep-related health status from both patient and bed partner significantly improved, as assessed by the SOS, and the SBPS.

Isolated snoring in adults is a very common cause of distress for patients and their bed partners. Consequently, a high number of snorers seek medical help.

Different treatment modalities for primary snoring are available, ranging from conservative behavioural measures, such as weight loss and posture therapy to more or less invasive procedures. The latter most frequently aim to reduce soft tissue hypertrophy related with snoring—e.g., uvulopalatopharyngoplasty (UPPP), laser-assisted uvulopalatoplasty (LAUP) and radio-frequency tissue volume reduction (RFTVR)—and or to stiffen the soft palate by inducing scar tissue—e.g., pillar procedure, injection snoreplasty and radiofrequency. Most of these procedures are performed under local or general anaesthesia and are associated with (prolonged) postoperative pain and many potential side effects, such as pharyngeal dryness, globus sensation, vocal change, pharyngonasal reflux and even severe complications, such as bleeding and death [11–15]. On top of that, low success rates and a significant number of relapses have been described [11, 12, 14].

Mandibular advancement devices (MADs) also have gain interest in the treatment of OSAS. Worn intra-orally at night, MADs are attached to the teeth. Therefore, MAD's are restricted for patients with healthy dentition [16, 17]. Moreover, patients often decline to wear an oral appliance or discontinue such therapy due to the almost universal initial side-effects of excessive salivation, teeth and jaw discomfort [16, 18].

Non-invasive NightLase laser-therapy might tackle these shortcomings. The treatment, performed without any anaesthesia, was very well tolerated by all patients. All the observed side effects were (very) mild and transient, with very mild sore throat (in 20% of patients) and a very mild sensation of a dry throat (in 15% of patients) at day 1 after treatment being the most often reported side effects. No severe adverse effects occurred and none of the patients developed ulceration or scarring of the oral mucosa. These findings are consistent with literature. Pooled data from published studies, including 294 patients, showed that the most common side effect is a transient dry throat and foreign body sensation, which is present in up to 19% of patients [7]. 4% of patients reported transient altered palatal sensation, while 1% of patients reported sore throat and aphthous ulcer formation, respectively. No serious adverse effects or scarring were reported in any of the published studies with the NightLase protocol [2–7].

The results of our study are promising, but still not all subjects reported an adequate reduction of snoring. The small sample size and the short follow-up limits the significance of our conclusions. Nevertheless, our results are in accordance with available literature on NightLase. Fini Storchi et al. found in a prospective study (*n* = 40) a satisfaction rate of 85%, which was sustainable at 20 months in 71.2% of patients [4]. Retrospectively, Cetinkaya et al. found an average rate of improvement after three Er:YAG treatments of 65% (*n* = 33) [2]. The greatest improvement and satisfaction were experienced by patients aged ≥ 50 years. They suggested that variations in response to the treatment might be related to the difference in the collagen remodelling capacity of each patient [2].

The efficacy of NightLase was comparable with the results of a randomised controlled study investigating the efficacy of temperature-controlled radiofrequency ablation in 26 non-sleepy snorers compared to sham surgery [11]. The authors reported that snoring estimated by a bedroom partner on a VAS scale from 0 to 10, was reduced by surgery compared with sham surgery from a mean of 8.1 to 5.2 and by sham surgery from 8.4 to 8.0. Similarly, Ferguson et al. randomized 46 snoring patients with an apnea–hypopnea index between 10 and 27 to laser-assisted uvulopalatoplasty or no treatment, followed up for 7 months. Snoring intensity estimated by a bedroom partner on a VAS scale from 0 to 10 was reduced by surgery from a mean of 9.2 to 4.8 and by no treatment from 8.9 to 8.5 [19]. Larossa et al. randomized 28 snoring patients with an apnea–hypopnea index < 30 to laser-assisted uvulopalatoplasty or sham surgery and followed them for 3 months [20]. They found no significant difference in change in subjective snoring intensity, Snoring index or decibels of snoring [20].

These results indicate that the effectiveness of NightLase is comparable with that of more invasive treatments. Still, as mentioned above, not all subjects reported an adequate reduction of snoring. More large scale prospective and longterm studies are necessary. They will help us to identify the patients who most likely will benefit from this minimal invasive treatment.

## Conclusions

NightLase treatment significantly reduced snoring compared to sham treatment. Its efficacy is similar to that achieved with other more aggressive treatments.

Taking into account the minimal invasiveness of NightLase, this procedure seems to represent a valuable, low-risk treatment option for primary snoring/mild OSAS. More prospective large-scale and long-term follow-up studies are warranted to prove its (long-term) efficacy.
