# Correction: Treatment of snoring using a non-invasive Er:YAG laser with SMOOTH mode (NightLase): a randomized controlled trial

**DOI:** 10.1007/s00405-022-07624-z

**Published:** 2022-08-29

**Authors:** Valerie A. Picavet, Marc Dellian, Eckard Gehrking, Alexander Sauter, Katrin. Hasselbacher

**Affiliations:** 1 ENT, Praxis für Ästhetik/HNO, Ludwigstrasse 7, Augsburg, Bavaria Germany; 2 ENT, MVZ Moser Gehrking Sauter und Partner, Augsburg, Bavaria Germany; 3 ENT Praxis Hasselbacher-Picavet & Partner, Donauwörth, Bavaria Germany

## Correction: European Archives of Oto-Rhino-Laryngology https://doi.org/10.1007/s00405-022-07539-9

The article “Treatment of snoring using a non-invasive Er:YAG laser with SMOOTH mode (NightLase): a randomized controlled trial”, written by Valerie A. Picavet, Marc Dellian, Eckard Gehrking, Alexander Sauter, Katrin Hasselbacher, was originally published electronically on the publisher’s internet portal on 22 July 2022 without open access. With the author(s)’ decision to opt for Open Choice the copyright of the article changed on 15 August 2022 to © The Author(s) 2022 and the article is forthwith distributed under a Creative Commons Attribution 4.0 International License, which permits use, sharing, adaptation, distribution and reproduction in any medium or format, as long as you give appropriate credit to the original author(s) and the source, provide a link to the Creative Commons licence, and indicate if changes were made. The images or other third party material in this article are included in the article's Creative Commons licence, unless indicated otherwise in a credit line to the material. If material is not included in the article's Creative Commons licence and your intended use is not permitted by statutory regulation or exceeds the permitted use, you will need to obtain permission directly from the copyright holder. To view a copy of this licence, visit http://creativecommons.org/licenses/by/4.0/.

